# Double-balloon endoscopic retrograde cholangiography can make a reliable diagnosis and good prognosis for postoperative complications of congenital biliary dilatation

**DOI:** 10.1038/s41598-021-90550-7

**Published:** 2021-05-26

**Authors:** Chiyoe Shirota, Hiroki Kawashima, Takahisa Tainaka, Wataru Sumida, Kazuki Yokota, Satoshi Makita, Hizuru Amano, Aitaro Takimoto, Akinari Hinoki, Hiroo Uchida

**Affiliations:** 1 Department of Pediatric Surgery, Nagoya University Graduate School of Medicine, 65 Tsurumai-cho, Showa-ku, Nagoya, 466-8550 Japan; 2 Department of Endoscopy, Nagoya University Hospital, Nagoya, Japan

**Keywords:** Choledocholithiasis, Paediatric research

## Abstract

Bile duct and anastomotic strictures and intrahepatic stones are common postoperative complications of congenital biliary dilatation (CBD). We performed double-balloon endoscopic retrograde cholangiography (DBERC) for diagnostic and therapeutic purposes after radical surgery. We focused on the effectiveness of DBERC for the treatment of postoperative complications of CBD patients. Bile duct and anastomotic strictures and intrahepatic stones are common postoperative complications of congenital biliary dilatation (CBD). We performed double-balloon endoscopic retrograde cholangiography (DBERC) for diagnostic and therapeutic purposes after radical surgery. We focused on the effectiveness of DBERC for the treatment of postoperative complications of CBD patients. This retrospective study included 28 patients who underwent DBERC (44 procedures) after radical surgery for CBD between January 2011 and December 2019. Strictures were diagnosed as “bile duct strictures” if endoscopy confirmed the presence of bile duct mucosa between the stenotic and anastomotic regions, and as “anastomotic strictures” if the mucosa was absent. The median patient age was 4 (range 0–67) years at the time of primary surgery for CBD and 27.5 (range 8–76) years at the time of DBERC. All anastomotic strictures could be treated with only by 1–2 courses of balloon dilatation of DBERC, while many bile duct strictures (41.2%) needed ≥ 3 treatments, especially those who underwent operative bile duct plasty as the first treatment (83.3%). Although the study was limited by the short follow-up period after DBERC treatment, DBERC is recommended as the first-line treatment for hepatolithiasis associated with biliary and anastomotic strictures in CBD patients, and it can be safely performed multiple times.

## Introduction

Radical surgery for congenital biliary dilatation (CBD) is often performed in childhood, and children live for decades after undergoing this procedure. Bile duct strictures and anastomotic stricture, as well as the associated intrahepatic stones, are the most common long-term complications following radical surgery for CBD, with an incidence of 2.7–11%^1–3^. These late complications often begin more than 10 years after radical surgery and are frequently treated only in adulthood, even if the surgery is performed in childhood^3^. Therefore, to understand the prognosis of CBD, it is necessary to examine the long-term complications, including those in adults, instead of focusing only on childhood treatment.

The better best treatment option for complications that cause bile stasis, including bile duct strictures and anastomotic strictures, remains debatable. In the past, additional surgeries such as bile duct plasty and hepatectomy were the main treatment options, and some patients were subjected to a long series of repeated operations^4,5^. Although percutaneous transhepatic cholangiodrainage (PTCD) in some puncturable cases is available as a treatment option, it is time-consuming and highly burdensome for patients.

Double-balloon enteroscopy (DBE) was introduced in 2003, with double-balloon endoscopic retrograde cholangiography (DBERC) for the postoperative reconstructed intestinal tract being reported by Haruta et al. in 2005^6^. Treatment with DBERC has evolved greatly since then, having been used in patients with reconstructed intestinal tracts after procedures, such as liver transplantation and surgery for bile duct cancers^7–10^. However, the outcomes in CBD patients have not been clearly documented except in case series^11,12^. We performed DBERC for both diagnostic and therapeutic purposes in both pediatric and adult patients after radical surgery for CBD. Before the introduction of DBERC, we could not determine whether the cause of intrahepatic stones was bile duct strictures or anastomotic strictures. One of the reasons CBD is not a simple disease that can be relieved only by radical surgery is that there are cases of difficult-to-treat strictures in the intrahepatic bile duct^1,3–5^. Hepatectomy may eventually be required for such complicated cases of bile duct strictures. Therefore, in determining the best treatment strategy, it is very important to determine whether the cause of postoperative intrahepatic stones is a bile duct stricture or an anastomotic stricture.

In this study, we aimed to determine the effectiveness of DBERC for hepatolithiasis associated with biliary and anastomotic strictures among pediatric and adult CBD patients.

## Results

DBERC was performed 44 times in 28 patients (4 males and 24 females) after primary (radical) surgery for CBD. Eleven of these patients underwent primary surgery at our hospital while the remaining 17 underwent surgery at other hospitals. The median age of patients at the time of primary surgery for CBD was 4 years (range 0–67 years; n = 28), and the average age at the time of DBERC was 27.5 years (range 8–76 years; n = 44). The median duration from primary surgery to DBERC was 17 years (range 0–48 years). In 7 cases, surgical records or medical information sheets confirmed that bile duct plasty was performed at the time of primary surgery (Cases 5, 7, 13, 14, 15, 16 and 17).

In 3 of the 28 patients (3 of the 44 procedures), an endoscope could not be advanced through the intestine owing to adhesions or intestinal stenosis. In the remaining 25 patients, the procedure was only performed to diagnose cholangiocarcinomas in two patients and DBERC was performed to treat bile duct strictures, anastomotic strictures, and both types of strictures in 15, 6, and 2 patients, respectively (Table 1). The reason for performing DBERC in 23 patients was cholangitis in two patients, intrahepatic bile duct dilatation or stones suspected on imaging in 19 patients, elevated transaminase levels without symptoms in one patient, and atrophy of the right hepatic lobe in one patient.

**Table 1:** Characteristics of patients who underwent double-balloon endoscopic retrograde cholangiography (DBERC) between 2011 and 2019 in our facility.

Variable	Value (28 patients, 44 operations)
Sex	24 females, 4 males
Age (years)	27.5 (8–76)
Age at first surgery (years)	4 (0–67)
Time from first surgery to DBERC (years)	17 (0–48)
DBERC not feasible	3 (11%)
Bile duct strictures	15 (54%)
Anastomotic stricture	6 (21%)
Bile duct and anastomotic strictures	2 (7%)
Diagnostic purpose	2 (7%)

Data are presented as the median (range) or number (percentage).

**Figure 1:**
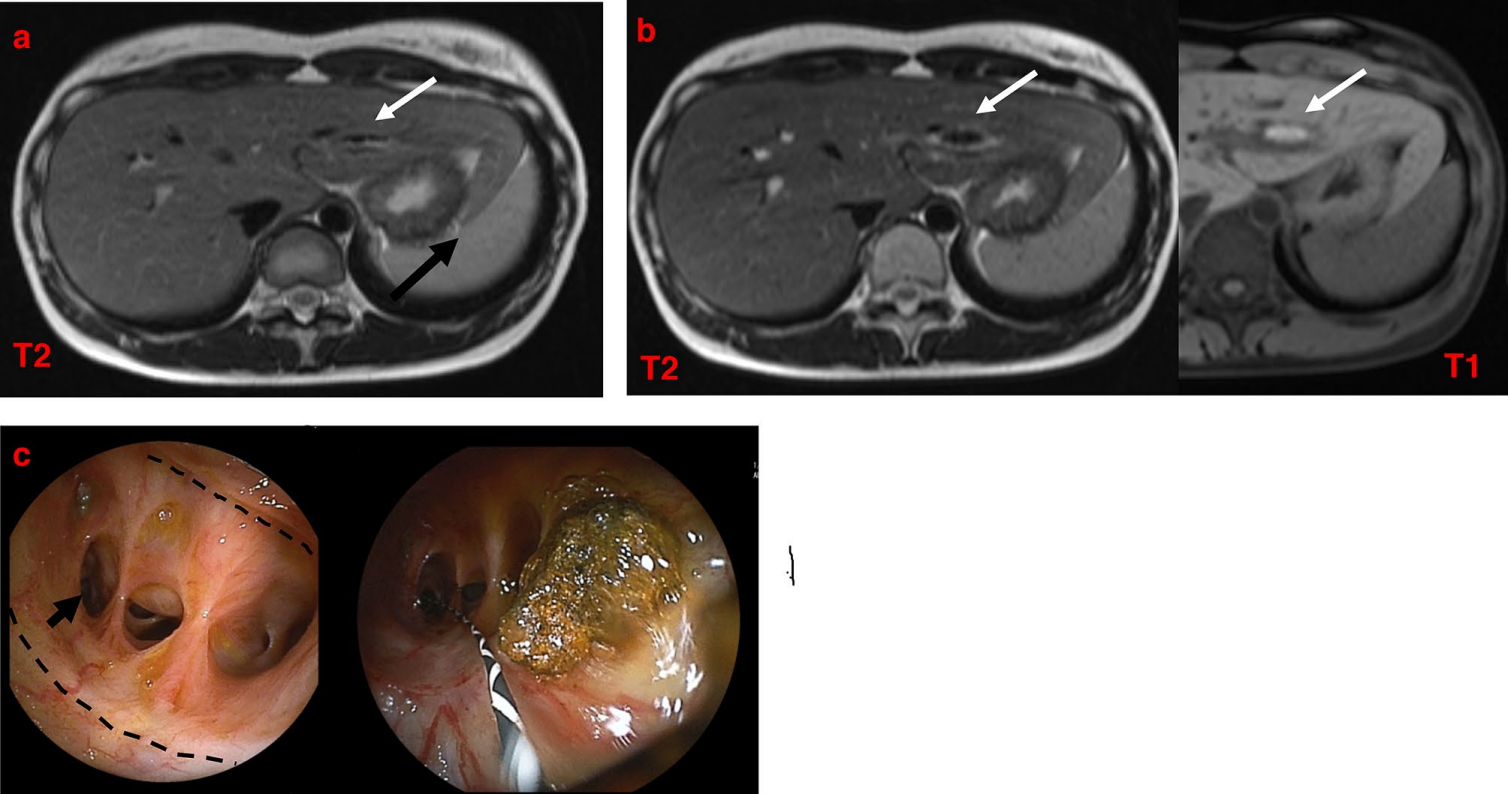
Case 6 from Table 1. (**a**) No abnormalities are seen in MRCP 20 years after radical surgery. (**b**) Twenty-five years after radical surgery, MRCP identifies intrahepatic bile duct dilatation and stones. (**c**) Bile duct mucosa is found in front of the stenosis. There is no stenosis at the anastomosis (dotted line). The stricture is noted in the second part of the bile duct. (arrow) Lithectomy is performed at DBERC. *DBERC* double-balloon endoscopic retrograde cholangiography, *MRCP* magnetic resonance cholangiopancreatography.

Patients with bile duct strictures were treated by balloon dilation of the stenotic area and/or hepatolithectomy (Table 2). Our hospital began performing DBERC on patients after surgery for CBD in December 2011. Prior to that, patients who developed bile duct and anastomotic strictures would undergo additional operative bile duct plasty and re-anastomosis. After the introduction of DBERC, all patients with postoperative bile duct or anastomotic strictures underwent DBERC. Six of these patients (Cases 1–6) underwent additional surgery (operative bile duct plasty) after the primary surgery followed by further DBERC. Two patients (Cases 1 and 2) underwent DBERC for bile duct strictures after two operative bile duct plasties, and they have been asymptomatic for 23 and 19 months after DBERC, respectively. Of the remaining four patients (Cases 3–6) who underwent DBERC after operative bile duct plasty, two did so several times but eventually required hepatectomy owing to residual hepatolithiasis (Cases 4 and 5), and one of them (Case 4) underwent DBERC again because of bile duct stones (B3) after hepatectomy (right lobectomy). One patient was scheduled for hepatectomy after DBERC due to complete obstruction of the right hepatic duct (Case 3). Another patient (Case 6) underwent radical surgery at the age of four years and another operation for bile duct plasty at the age of eight years (Table 2). Subsequently, she was asymptomatic and there were no abnormalities on MRCP 20 years after radical surgery. Twenty-five years after radical surgery, MRCP identified intrahepatic bile duct stones. We performed DBERC, diagnosed her with bile duct stricture and stones, and performed lithotomy (Fig. 1). The first six cases, except case 3, had been asymptomatic for more than a year after the last treatment (Table 2).

**Table 2:** Treatment history of 15 patients with bile duct strictures.

Case	Treatment 1	POY*	Treatment 2	POY*	Treatment 3	POY*	Treatment 4	POY*	Treatment 5	POY*	Years from last treatment	Symptom	Site of stenosis
1	Operative BDP**	0.5	Operative BDP**	1.0	**DBERC**	48					1.9	−	Right hepatic duct
2	Operative BDP**	6.1	Operative BDP**	6.8	DBERC^D^	23					1.6	−	Anterior branch
3	Operative BDP**	8.0	DBERC^**F**^	16	Hepatectomy(planned)						5.5	+ ***	Right hepatic duct
4	Operative BDP**	11	DBERC^**F**^	40	DBERC^**F**^	40	Hepatectomy	43	**DBERC**	45	1.7	−	B3 B8
5^+^	Operative BDP**	3.6	DBERC^**F**^	5.3	DBERC^**F**^	6.6	DBERC^**F**^	6.6	Hepatectomy	9.6	1.5	−	Posterior branch
6	Operative BDP**	4.4	**DBERC**	25							3.2	−	B2
7^+^	DBERC^**F**^	14.7	Hepatectomy	15							1.2	−	Left hepatic duct
8	**DBERC**	19	DBERC^−^	19	DBERC^−^	21					3.8	−	B2 B3
9	DBERC^**F**^	22	DBERC^**F**^	24	**DBERC**	25					3.2	−	B3
10	**DBERC**	19	DBERC^−^	20							0.2	−	Left hepatic duct
11	DBERC^−^	15	**DBERC**	18							1.6	−	B5
12	**DBERC**	7.3	**DBERC**	12							0.4	−	B5 B6
13^+^	**DBERC**	16									0.9	−	Left hepatic duct
14^+^	**DBERC**	0.7									5.4	−	B6
15^+^	**DBERC**	1.8									0.5	−	Posterior branch

Superscripted plus sign (^+^) beside the case number: bile duct plasty was performed at the time of the primary operation.

**DBERC**: Complete lithotripsy.

DBERC^−^: No stones and stenosis.

DBERC^**F**^: Residual stones after DBERC.

DBERC^D^: Stenosis without stones.

*POY: postoperative years.

**BDP: bile duct plasty.

***complete obstruction of the right hepatic duct.

DBERC, double-balloon endoscopic retrograde cholangiography.

After December 2011, nine patients (Cases 7–15) underwent DBERC as the first-line treatment for bile duct strictures following primary surgery. One patient underwent hepatectomy after DBERC-assisted hepatolithectomy was deemed infeasible due to complete occlusion of the left hepatic duct (Case 7). Three patients underwent DBERC twice, and two underwent the procedure thrice for residual hepatolithiasis and/or restenosis. The median observation period from the last treatment was 14 months (range 2–26). None of the nine patients had residual hepatolithiasis (Table 2).

Two patients developed both bile duct and anastomotic strictures. Their conditions improved following balloon dilation of both areas, as well as hepatolithectomy (one operation per patient), and they were carefully monitored for restenosis. These two patients were followed-up for 42 and 82 months, respectively, after DBERC with no recurrence (Table 3).

**Table 3:** Treatment history of two patients with anastomotic and bile duct strictures.

Case	Treatment 1	POY*	Years from last treatment	Symptom	Site of stenosis
16^+^	**DBERC**	24	3.5	–	Right and left hepatic duct
17^+^	**DBERC**	12	6.8	–	Right and left hepatic duct

**DBERC**: complete lithotripsy.

*POY: post radical operative years.

DBERC, double-balloon endoscopic retrograde cholangiography.

**Figure 2:**
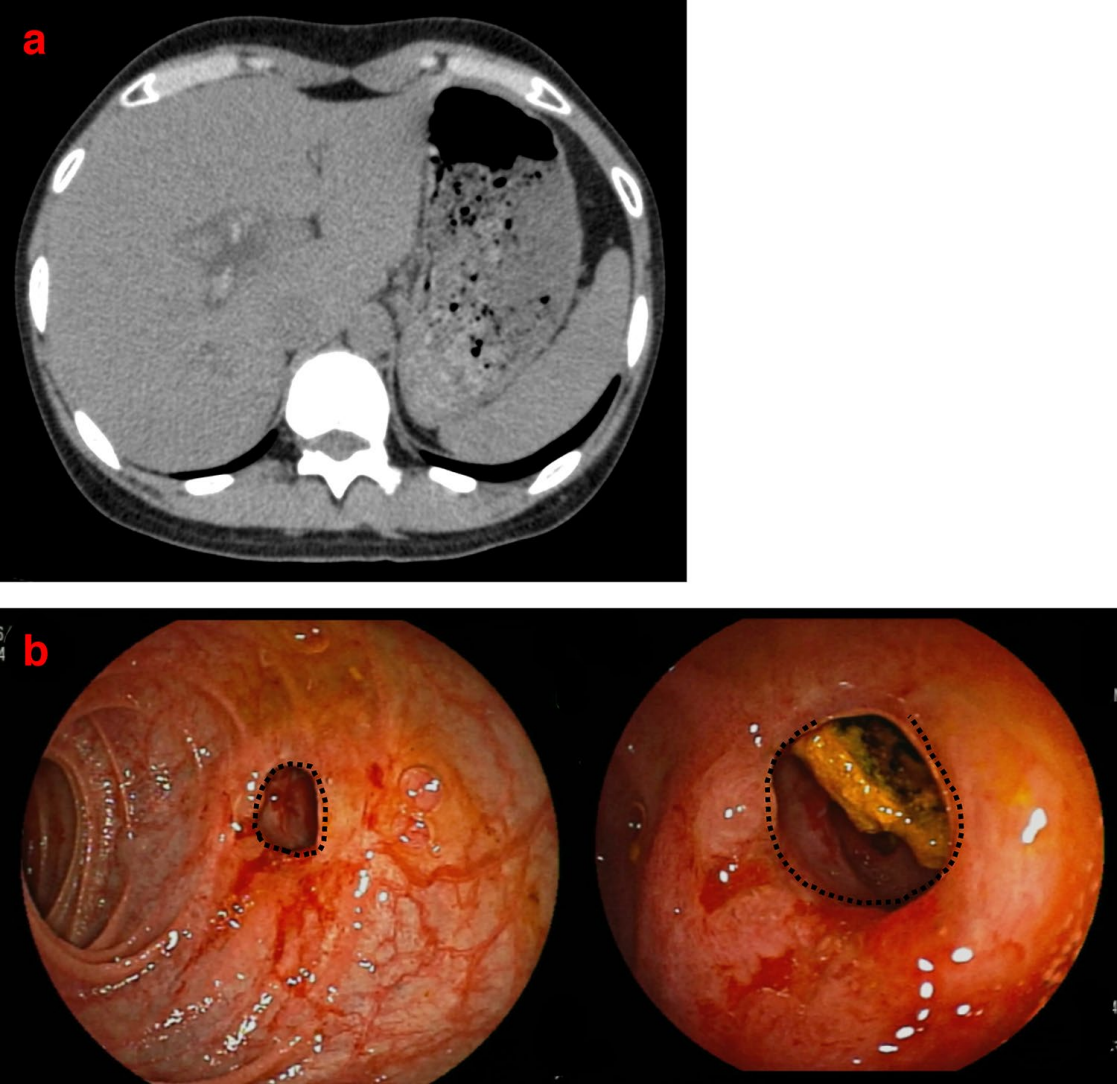
Case 3 from Table 4. (**a**) Stones are identified on computed tomography twelve years after radical surgery. (**b**) An anastomotic stricture was identified using DBERC. (dotted line) Balloon dilation and lithectomy were performed. *DBERC* double-balloon endoscopic retrograde cholangiography.

Six patients with anastomotic strictures in the absence of bile duct strictures were treated by balloon dilation of the affected region and/or lithectomy, and symptoms improved in all patients. Six patients were followed-up for 2–98 months after DBERC with no recurrence (Table 4). One patient (Case 20) underwent primary surgery at the age of 23 years and was asymptomatic for over 10 years. Twelve years after radical surgery, abdominal pain occurred, and stones were identified on computed tomography. Anastomotic stenosis was confirmed by DBERC, and lithotomy was performed (Fig. 2). In 6 (cases 3, 4, 5, 7, and 9 in Tables 2 and case 18 in Table 4) of the 23 cases, not all the stones were removed after a single DBERC. In two cases, the stones were completely removed after multiple DBERCs. One case was a B3 stenosis (Table 1, case 9). Another case was an anastomotic stenosis, and the stones remained in the left hepatic duct (Table 4, case 18). Seventeen (74%) of the lumps were removed with a single DBERC. The stones were confirmed to have been completely removed by contrast imaging. In addition, follow-up MRCP revealed no evidence of residual stones.

**Table 4:** Treatment history of six patients with anastomotic strictures.

Case	Treatment 1	POY*	Treatment 2	POY*	Years from last treatment	Symptom
18	DBERC^**F**^	1.2	**DBERC**	1.3	3.4	–
19	**DBERC**	15			8.2	–
20	**DBERC**	12			4.0	–
21	**DBERC**	5.3			1.2	–
22	**DBERC**	4.5			6.1	–
23	**DBERC**	1.7			0.2	–

Superscripted plus sign (^+^) beside the case number: bile duct plasty was performed at the time of the primary operation.

**DBERC**: Complete lithotripsy.

DBERC^**F**^: Residual stones after DBERC.

*POY: postoperative years.

DBERC, double-balloon endoscopic retrograde cholangiography.

Two patients developed DBERC-related complications, one had ascending jejunal limb stenosis 5 years after surgery (at the age of 8 years), and the other had intussusception 12 years after surgery (at age 53 years). One of these patients underwent DBE thrice to treat ascending jejunal limb stenosis and later underwent laparoscopy-guided reconstruction of the upper jejunum. In the other patient, intussusception was treated using DBE.

## Discussion

Late postoperative complications of CBD include intrahepatic stones, intrapancreatic residual bile duct, and bile duct cancer^2^. The incidences of intrapancreatic residual bile duct and bile duct cancer have been reported to be low^1,13,14^. Conversely, the most common late complication after CBD surgery is intrahepatic stones due to bile duct strictures and/or anastomotic strictures. Intrahepatic stones due to intrahepatic bile duct strictures after CBD surgery are difficult to treat^1,3–5^. Indeed, postoperative complications due to bile duct strictures were difficult to treat in our study. Moreover, before the introduction of DBERC, the main treatment options for bile duct strictures were reoperations, such as operative bile duct plasty or hepatectomy. However, the therapeutic effect of reoperations was not feasible^1,5,15^. Currently, DBERC is a treatment option in such cases, and in cases where DBERC is ineffective, it can be repeated multiple times. In addition, the burden associated with DBERC on the patient is relatively low^16^. Twelve patients in our case series underwent DBERC multiple times, and hepatectomy was deemed necessary for three patients. This decision was made based on DBERC findings in each case, illustrating the usefulness of this approach in selecting treatment plans.

DBERC failure was noted in three cases, including a 39-year-old (36 years following surgery), a 52-year-old (24 years following surgery), and a 76-year-old (28 years following surgery). The reported complications of DBE and DBERC include gastrointestinal perforation, hemorrhage, damage to the intestinal mucosa or bile ducts, cholangitis, and pancreatitis; however, these complications are infrequent, observed in only 3–7% of cases, and have been shown to resolve after conservative treatment^16–18^.

We observed two complications in our case series, each affecting one patient: (1) upper jejunal stenosis and (2) intussusception. Jejunal stenosis developed in the child following treatment for anastomotic stricture. DBERC procedure took longer than usual in this case because it was difficult to pass the endoscope through to the anastomotic region, which may have contributed to the upper jejunal stricture. In the other patient, intussusception was repaired by DBE.

It is important to review surgeries when complications occur in order to improve surgical results. However, in cases of CBD, where the interval between radical surgery and treatment of complications is long, complications are treated regardless of the findings of radical surgery. Moreover, there is a possibility that the experience gathered from treating complications may not be relevant in improving radical surgery. In carrying out this retrospective study, we clearly defined and distinguished diagnoses of "anastomotic stricture” and "bile duct strictures" that had been vaguely recorded at the time of DBERC. This is because "anastomotic strictures” and “bile duct strictures” are different conditions. Anastomotic strictures are due to technical problems at the anastomosis of the bile duct and the intestine, while bile duct strictures are caused by patient-specific anatomical conditions, such as membranous stenosis and septal stenosis of the intrahepatic bile duct^19^. Bile duct plasty can be added to bile duct strictures, but its long-term outcomes are not clear. In this study, we clearly defined "anastomotic strictures" and "bile duct strictures", and we hope our definition will make it possible to provide accurate feedback for radical surgery.

Patients in our study underwent DBERC at a median time of 17 years after their first operation for CBD, indicating the need for long-term follow-up among those with this disease, even after surgical intervention. In this study, anastomotic strictures could be treated by just 1–2 balloon dilatations during DBERC, and a second surgical operation was not necessary. On the other hand, it is difficult to treat bile duct strictures, and intrahepatic stones that occur even after operative bile duct plasty. Based on our treatment results, DBERC might be more effective than operative bile duct plasty. In cases of bile duct strictures, recurrence of intrahepatic stones after balloon dilatation of DBERC may necessitate hepatectomy.

This study has some limitations. First, since we have a short duration of observation after the induction of DBERC treatment; therefore, decisive conclusions could not be drawn. Second, CBD patients with bile duct strictures or anastomotic strictures account for less than 10% of CBD and cannot be statistically evaluated^1–3^. However, in this study, performing DBERC for bile duct strictures or anastomotic strictures was a new treatment strategy, and if it was infeasible, hepatectomy rather than bile duct plasty was performed. By accurately diagnosing bile duct strictures and anastomotic stricture using DBERC, it is possible to correctly evaluate the indication of hepatectomy for recurrent stones.

DBERC is an effective first-line modality for the treatment of hepatolithiasis and to differentiate bile duct strictures and anastomotic strictures. DBERC can be safely performed multiple times and the decision of treatment strategy. Successful DBERC-based therapy prevents repeated surgery and might improve patients’ outcomes. Our study demonstrates the importance of prompt DBERC and careful follow-up after radical surgery for CBD. DBERC can be the main treatment strategies for cholestasis and hepatolithiasis, which are the most common long-term complications of CBD.

## Methods

This retrospective study was approved by the institutional ethics board of Nagoya University Hospital (approval number: 2019-0417) and was performed according to the guidelines described in the Helsinki Declaration for biomedical research involving human patients. Since this was a retrospective observational study and the data analyzed were anonymized, informed consent from participants or their parents/guardians was obtained through an opt-out method on our hospital website in accordance with the Ethical Guidelines for Medical and Health Research Involving Human Subjects in Japan. Records were reviewed for all patients who underwent DBERC for the treatment of postoperative complications of CBD at our hospital from January 2011 to December 2019. Patients were enrolled if they had undergone DBERC after radical surgery to treat CBD at our hospital or had presented at our facility after undergoing such an operation at another hospital. In our institution, postoperative long-term follow-up was done at least once a year, during which blood tests and ultrasonography were done. Magnetic resonance cholangiopancreatography (MRCP) was performed once every 5 years, even if there are no symptoms. If symptoms or imaging tests suggests intrahepatic stones or bile stasis, DBERC is performed.

### DBERC procedure

DBERC was performed using a short-type double-balloon endoscope with an EI-530B endoscope (effective length: 1520 mm, working channel: 2.8 mm, FUJIFILM, Tokyo, Japan) or EI-580BT endoscope (effective length: 1550 mm, working channel: 3.2 mm, FUJIFILM) and TS13101 over-tube (FUJIFILM). CO_2_ insufflation was used in all procedures. After reaching the anastomosis, the body position was changed to dorsal or abdominal to perform ERC. For insertion into the bile duct, a 3.5 Fr catheter (PR-110Q-1, Olympus Medical Systems, Tokyo, Japan) or a 3.9 Fr catheter (TRUEtome™, Boston Scientific, Natick, MA, United States) with a 0.025-inch guidewire (VisiGlide2™, Terumo, Tokyo, Japan, or RevoWave-SJ™, Piolax Medical Devices, Kanagawa, Japan) was mainly used. A balloon catheter (ZARA™, Century Medical, Tokyo, Japan, or CRE™ PRO GI Wireguided, Boston Scientific) was used to dilatate the strictures. Extraction of stones was performed using a basket for stone extraction (FlowerBasket™, Olympus Medical Systems) and a balloon catheter (Multi-3V™ Plus, Olympus Medical Systems).

### Diagnosis of strictures

**Figure 3:**
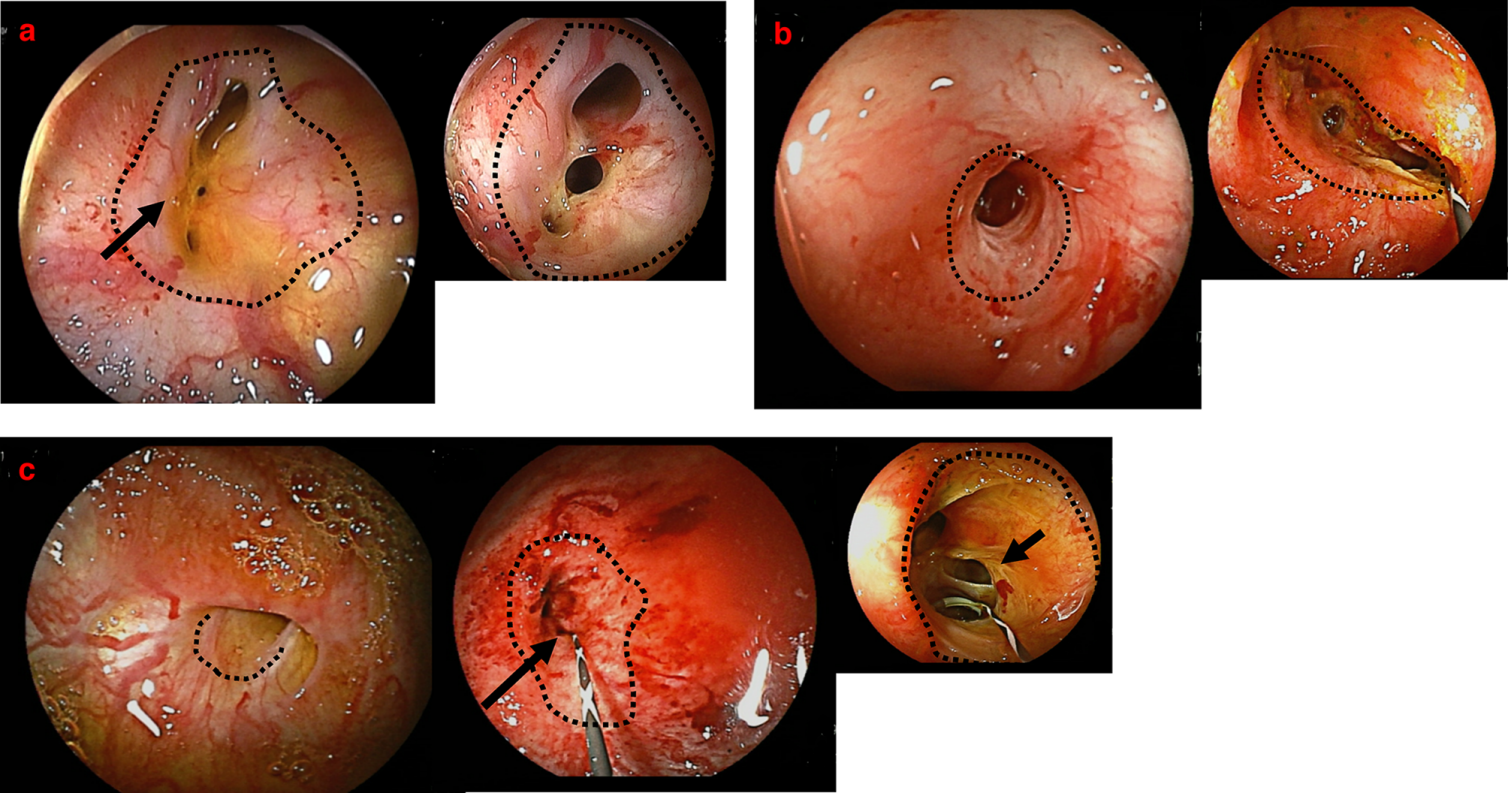
(**a**) Strictures diagnosed as “bile duct strictures.” No strictures at the anastomosis (dotted line) and bile duct mucosa (arrow) are found between the stenotic and anastomotic regions. The figure on the right shows the bile duct after balloon dilatation of strictures (narrowed bile duct) with DBERC. (**b**) Stricture diagnosed as an “anastomotic stricture.” The site of anastomosis is indicated with a dotted line. There is no bile duct mucosa between the stenotic and anastomotic regions. The figure on the right shows the bile duct after balloon dilation of the stenotic anastomosis. There are no strictures at the bile duct. (**c**) Strictures diagnosed as “anastomotic and bile duct strictures.” The anastomosis site is indicated with a dotted line. There is no bile duct mucosa between the stenotic and anastomotic regions. The stricture was diagnosed as an “anastomotic stricture.” The figure in the middle shows the bile duct after dilation of the anastomosis, bile duct strictures (arrow) are also seen. The figure on the right shows the bile duct after balloon dilation of the narrowed segment of the bile duct (arrow). *DBERC* double-balloon endoscopic retrograde cholangiography.

Strictures were diagnosed as “bile duct strictures” if the presence of bile duct mucosa between the stenotic and anastomotic regions confirmed on endoscopy, and as “anastomotic strictures” if the mucosa was not present (Fig. 3).

## Data Availability

All data from this study are not publicly available due to compliance to privacy. Summaries are available from the corresponding author on reasonable request.
